# What is learned from Mindfulness Based Childbirth and Parenting Education? – Participants’ experiences

**DOI:** 10.1186/s12884-018-2098-1

**Published:** 2018-12-03

**Authors:** Gunilla Lönnberg, Eva Nissen, Maria Niemi

**Affiliations:** Department of Public Health Sciences, Karolinska Institutet, Tomtebodavägen 18, 171 76 Stockholm, Sweden

**Keywords:** Mindfulness, Antenatal, Postpartum, Childbirth, Parenting programme, Stress, Anxiety, Depression, Experience

## Abstract

**Background:**

In the search for effective interventions aiming to prevent perinatal stress, depression and anxiety, we are evaluating a Mindfulness Based Childbirth and Parenting (MBCP) Program. In this study we explore the participants’ experiences of the program.

**Method:**

This is a descriptive qualitative study with influences of phenomenology. The participants were expectant couples who participated in the program and the pregnant women had an increased risk of perinatal stress, anxiety and depression. Ten mothers and six fathers were interviewed in depth, at four to six months postpartum. Thematic analysis of the transcripts was conducted.

**Results:**

The participants’ descriptions show a variety in how motivated they were and how much value they ascribed to MBCP. Those who experienced that they benefitted from the intervention described that they did so at an intra-personal level–with deeper self-knowledge and self-compassion; and on an inter-personal level–being helpful in relationships. Furthermore, they perceived that what they had learned from MBCP was helpful during childbirth and early parenting.

**Conclusion:**

Our findings demonstrate that most of the parents experienced MBCP as a valuable preparation for the challenges they met when they went through the life-changing events of becoming parents. The phenomenon of participating in the intervention, integrating the teachings and embodying mindfulness seems to develop inner resources that foster the development of wisdom.

**Trial registration:**

ClinicalTrials.gov ID: NCT02441595, May 4, 2015.

**Electronic supplementary material:**

The online version of this article (10.1186/s12884-018-2098-1) contains supplementary material, which is available to authorized users.

## Background

Depression and anxiety among young adults in Sweden are increasing [1] and among pregnant women the prevalence rate for depressive symptoms, indicative of possible depression is 13,7% [2]. It is urgent to address this problem among parents since perinatal stress, anxiety and depression do not only affect the mother or father. In the long-term these conditions may also affect the child’s mental and physical health [3, 4]. There are numerous pathways through which mental health is transferred from parent to child: During pregnancy, a mother’s levels of stress or negative affect impact the foetus and are risk-factors for disorders later in life [5, 6]. Once born, mother-infant attachment plays an important role for the child’s cognitive and emotional development as well as mental health later on [7]. If the parents are depressed or distraught they may not be sensitive to the child’s signals, and the attachment style may thus be less optimal. An insecure attachment style in infancy increases the risk of anxiety and behavioural disorders later in life [8]. Considering the important role that parents’ mental health plays for the children’s future mental health, and for the escalating mental health problem in our society, research in this area is well motivated [9].

Mindfulness Based Interventions (MBIs) such as Mindfulness Based Stress Reduction and Mindfulness Based Cognitive Therapy are widely used in health care settings and have shown to reduce the risk of depression relapse, alleviate chronic pain, anxiety and stress related health problems [10–12]. Mindfulness is a practice as well as a state of awareness. The practice consists of steering ones attention (commonly to the sensations of breathing) and cultivating an attitude of patience, acceptance, non-striving and non-judging. The practice aims at an awareness that makes it possible to have pleasurable as well as painful experiences without being reactive and running the risk of behaving in destructive ways, but rather being discerning and compassionate.

The mechanisms set in motion by mindfulness practice are multiple: An increased capacity for regulating attention makes it easier to shift focus, to notice more positive elements in ones experience and to step out of rumination. Building the capacity to observe experiences without reactivity leads to a decrease in negative affect, and the capacity for reappraisal and self-compassion increases. [13]

Newer versions of MBIs specifically tailored for expectant parents are beginning to show promising results regarding perinatal mental health [14, 15]. In the systematic review by Taylor et al. the qualitative findings are summarized as follows “participants reported that they thought they benefited from connecting with others within a group context, and thought that they had become more able to focus on the present moment, to regulate their negative responses to difficult situations, and to become more accepting of current experiences” [14].

We are currently evaluating the effects of an MBI called Mindfulness Based Childbirth and Parenting (MBCP) in a randomized controlled trial (RCT). In this qualitative study we aimed to gain a deeper understanding of the participants’ experiences of MBCP, to obtain insights into the acceptability of the intervention and into if–and in what ways–first-time parents found the intervention useful during their everyday living going through pregnancy, childbirth and early parenting.

## Methods

### Setting and sample

The intervention took place in Stockholm, Sweden and was targeted to pregnant women at risk of perinatal depression. Primiparous women and their partners were invited to the RCT. They were informed that the aim of the study was to compare and evaluate two different parenting programs (MBCP and a Lamaze course) and that they were going to be randomly assigned to one of them. Interested women were screened with a short questionnaire in order to select those who perceived high levels of stress and/or had a history of mental illness and/or some childhood adversity. Previous experience of mindfulness and on-going severe mental illness were exclusion criteria.

For this qualitative study consecutive sampling was used. At three months post-partum we asked the first ten couples who had attended the MBCP program, if they agreed to be interviewed. They were informed that the interview would be about their experiences of participating in the MBCP program as well as about their experiences of being pregnant/being an expectant father, the childbirth and the early parenting period.

### Intervention

The intervention was a slightly adapted version from the original MBCP program developed by Bardacke [16], with eight 2 h 15 min weekly sessions and a reunion after the babies were born, and it was taught by the first author. The program integrates the teaching of mindfulness with education regarding psychobiological processes during pregnancy, childbirth, breastfeeding, postpartum adjustments and the needs of an infant. Practices include body scan, mindful movement, sitting meditation, walking meditation, loving kindness meditation and insight dialogue, which is a dialogue exercise for couples specific for the MBCP program, where mindful speaking and listening are explored. Also, methods to meet pain (using ice-water), awareness of the baby, awareness of the breath and informal meditation in daily life are taught. Participants are asked to practice at home for 30 min per day in between sessions supported by audio-files with guided instructions and informative texts.

### Data collection and analysis

The second author, a neutral person in the eyes of the participants, conducted the interviews in the participants’ homes at between 4 and 6 months postpartum. The interviews were in-depth, and conducted with the mother and father individually. They began with an open question regarding their experience of the pregnancy period. Further on, probes were used to explore their experiences of MBCP as well as of childbirth, care of their baby, breastfeeding and coping with challenges (see interview guide, Additional file 1). The interviews were recorded and transcribed verbatim by the first author. The text was analysed using thematic analysis according to Braun and Clarke [17] with a phenomenological inductive approach [18]. After repeated readings of the text, as well as making mind-maps and taking notes, meaning units were highlighted and initial codes were generated. As the codes developed, and meaning units were coded accordingly, patterns were successively identified that allowed the grouping of codes into initial themes. The themes were reviewed and more carefully defined and named as the analysis progressed. Translation of the quotes was conducted by both first and second author separately, then matched and agreed on for the final version.

Regarding the first author’s pre-understanding of mindfulness, both from personal experience and as a teacher of MBCP, “bridling” (a reflective stance) was required to be able to focus on an analysis that was data-driven [19]. This process was facilitated by the two co-authors overseeing and discussing the analysis. Pre-understanding can also enrich the process, as Merleau-Ponty pointed out: When we connect past experience with present moment it can be a juncture where new and expanded knowledge is made possible [20].

## Results

### Participant characteristics

Of the ten couples asked to be interviewed, all but 4 fathers agreed. The age of the participants ranged from 23 to 43 years. All but two of them had university degrees, most of them worked full time and they all had average or above average levels of income. All of them lived with their partner. They were Swedish-born, apart from four participants who were from Germany, Syria, Iran and Finland. Questionnaires filled out at baseline in the RCT showed that six of the participating mothers perceived high levels of stress; six of them had high scores on the depression questionnaire; and four had low self-efficacy for giving birth. Three of the fathers also perceived high levels of stress at baseline, and two scored high on the depression questionnaire. Seven of the mothers had uncomplicated vaginal deliveries. Mode of delivery for the other three was one vacuum extraction and two planned C-sections – one due to the baby being in breech position and the other due to fear of childbirth. In general, the mothers gave richer answers than the fathers, thus the amount of transcribed text was larger from the interviews with the mothers compared to interviews with the fathers.

### Findings

The participants’ descriptions of their initial attitudes toward MBCP and their motivation and engagement during the course are reported in the first theme: “Acceptability”. Furthermore, the participants spoke about finding new helpful ways of relating to themselves and the world due to participating in the MBCP program. Thus, the second theme is: “A new way of relating”. This theme is derived from four sub-themes:

1. Relating to oneself with greater insight and compassion
2. Help with coping with the pain and fear of childbirth
3. Help with challenges in caring for the baby
4. Relating to each other with more presence and patience

The themes are exemplified by quotes from the transcripts marked with M for mother and F for father.

#### Acceptability

We found that all of the mothers and half of the fathers had come to the course hoping to get some kind of help, ranging from wanting to decrease one’s fear of childbirth to wanting to increase one’s resilience in facing stress or negative affect. The majority of the mothers and fathers described having felt open and curious about the course before it had started. Those participants who both wished to get some kind of help and who also felt happy about having been randomized to MBCP were very motivated and engaged in the course. These same participants also ascribed a great value to MBCP at the time of the interview.

*Well, I have also thought ... that I might try out mindfulness in order to reduce my stress ... But I never got around to it, so I was very happy to get to participate in this course … I think it gave me a lot.* M

Three of the mothers described that before the course began they had felt hesitant that MBCP would be of any help to alleviate stress or anxiety. These participants ascribed some value to MBCP but they were not as enthusiastic as the other mothers. One of them described trying to overcome her disappointment with the randomization–she managed somewhat and engaged in the course, but also continued to feel some resistance:

*I had been hoping for the other course* (laughing) *because I think, I have always thought that mindfulness seems so airy-fairy, and always felt that it isn’t for me… And then I went to the first session of the course and it was a bit like I had thought, it was a bit too airy-fairy for me actually… I really wanted to give it a chance, and I think I did so too. … I did get something out of it.* M

One father described that he didn’t believe that the practice of mindfulness could be beneficial. He also described that he didn’t have any problems with stress. He explained that even thought he didn’t appreciate the course at all; he still attended the group sessions in order to support his partner. In contrast, statements from another father show that initial scepticism could change during the course.

*It eliminated some preconceived notions that I had had. I thought that these kinds of things were spaced-out, everything related to, well, yoga and such things, I had never understood them, not knowing what they were about. But now I know, with hindsight, that it is really, really positive ... I’m very happy about that.* F

There were indications that participating together as a couple influenced the acceptability in both directions, either strengthening or weakening it. The experience of MBCP was influenced by the partner’s experience, and with a partner who didn’t appreciate the course it’s possible that it was more difficult to overcome one’s own scepticism. As a contrast, there were also participants who ascribed value to the course due to the changes they experienced MBCP had brought about in their partner.

*(The course was) really good, especially for my partner, he changed completely. Because he used to be short tempered, ... But now he changed a lot in his behaviour ... and became calmer as a person and much more harmonious.* M

Participants reported enjoying the sessions and the homework as well as having difficulties with them. The most common challenges mentioned were time-constrains, falling asleep during the body scan, finding it difficult to focus as well as not remembering to do the home practices. Some participants also mentioned challenges like feeling impatient, feeling that practices such as loving kindness meditation or insight dialogue were awkward, and feeling sad because they had to skip the yoga practice due to physical pain. On the positive note the following was often mentioned: Doing the practices felt good–calming and clearing the mind, they enjoyed the relations with peers and the teacher, the aspect of self-care was valuable as well as becoming informed about childbirth and parenting. Most of the participants also appreciated participating with their partner.

The findings in this theme indicate that the combination of hoping to get some kind of help together with a preconceived notion that MBCP could provide this help, is the best prerequisites to experience MBCP as enjoyable and beneficial. The findings also show that a high level of scepticism or a feeling that the practice of mindfulness does not suit oneself can block the value of the course.

### A new way of relating

#### Relating to oneself with greater insight and compassion

The majority of the mothers and fathers described benefitting from the course due to changes in the way they relate to themselves. They described becoming aware of their own thinking processes and that this enabled them to steer their thoughts and behaviours in a more skilful way. The participants expressed this phenomenon by talking about an increased capacity to focus on the present moment–either the breath, the body or the task at hand. The increased ability to direct one’s attention was experienced as helpful for regulating emotions and coping with stress, pain, negative thoughts, insomnia, anxiety and rumination. They felt that it enabled them to calm down, put things in perspective and prioritize wiser.

*When you face a stressful moment or something that makes you, how should I say, upset or so, well, then you took a step back and thought “yes, but look at it from another perspective” ... It’s like you relax more.* F

Some participants managed to identify less with their thoughts, relating to thoughts as events in the mind, not necessarily true, letting them be and instead focusing on the task at hand with the baby:

*When a thought comes up, you don’t have to fight it, just let it be there… like I could think “but I’m a bad mother, why can’t I make the breastfeeding work” or something… but then I think that the thought can just be there, and I can just enjoy the moment, and then it [the thought] disappears..* M

Besides the ability to not identify with one’s thoughts, participants also described being able to cope with the discomfort of the moment, through reminding oneself of impermanence; that this too will pass. There were also participants who described being able to regulate their emotional reaction arising from physical pain, by focusing on the physical sensations instead of being carried away with anxious thoughts about the pain.

Participants described that the attitudinal foundation taught in MBCP was of importance and helped them be more resilient. These, in total eight attitudes, include for example non-judging, acceptance, curiosity and patience. The quote below is an example of how a participant embodied an attitude of curiosity toward experiences and non-judging toward her own reactions:

*In some ways the tolerance toward the surroundings increased ... You almost thought it was funny that you could look so matter-of-factly at your own irritation ... you shrugged your shoulders at it and then yea, you could move on.* M

An embodiment of acceptance and non-judging was described as influencing their way of dealing with difficult emotions. By accepting the emotions and not judging one-self for having them, it became possible to allow feelings to be felt instead of questioned.

The non-judging attitude was also perceived as a way of becoming more compassionate toward one-self, and this decreased negative affect. This was especially helpful for participants with habits of self-criticism. With increased self-compassion, there was also the willingness to accept that many things in life are beyond our control. Some experienced that the most valuable thing they learned from MBCP was to be kind to themselves while facing life’s uncertainties and the unknown and uncontrollable.

*I learned that especially with pregnancy, childbirth and children you can’t plan everything. Basically, there is a lot you can’t control so you should just accept it. Be kind to yourself… after all we are human..* M

The main findings in this sub-theme are thus that the informants experienced more calm, insight and self-compassion as they were able to steer their attention and adapt an approach of acceptance, non-judging and curiosity. The following sub-themes will describe the ways in which participants were helped by these new capacities during childbirth, early parenting and in the couple relation.

#### Help with coping with the pain and fear of childbirth

Participants talked about feeling more calm and secure in their role as parents-to-be, through the progression of the eight-week course. Some of them also described that MBCP reduced their fear of childbirth.

*From the beginning I was really scared of childbirth … (After the course) it felt like “I can do this!”* M

In labour, mindfulness practice was used for coping with pain and fear. It helped participants focus on the present moment and the breath during contractions, and in the pauses in between. It made it easier for them to accept things they had no control over, such as the pace of contractions, the time it took to give birth or if they didn’t get admitted to their hospital of choice and instead had to go to a hospital much further away. This acceptance of uncontrollable events was explained as coming from a sense of control over their own awareness, of being able to choose what to pay attention to and allow the process in the body to have its course. The ability to focus on the present moment was also helpful for coping with prolonged labour. Since they didn’t pay attention to time, their sense of time was changed.

*I guess I didn’t perceive that we had kept going for so long, I just took every moment as it came… It gives a sense of control to be able to focus on the breath… You’re not totally a slave to the pain or to what’s happening in the body, but you can choose to just let it happen.* M

One mother reported that as the intensity of labour increased she passed a limit.

*When the contractions started, all the way until I was about to push, it was an incredible help… But no, there simply was no coping strategy [while pushing]. It was to live or die. It just didn’t work any more.* M

Another mother had a similar experience as her labour became prolonged. This mother, as well as M1, experienced that there was a limit, and beyond that they could no longer use mindfulness as a way to cope. Five other mothers found mindfulness helpful all the way through the birth of their child. One of them spoke of being empowered. She explained this empowerment as coming from an attitude of curiosity–of meeting every contraction and every push–rather than trying to avoid it or escape from it.

*You wanted to be present and know how it feels. So you feel in a way quite strong and cool when giving birth to your child, like nature-strong in a way.* M

Many of the mothers mentioned the importance of the support from the partner during birth, and how MBCP had increased their partners’ confidence and knowledge in how to give support. Most fathers also mentioned feeling well informed and prepared by the course.

*I could give this good, this really, really, good support … if she lost it a bit I could simply remind her, lead her back on track again. Incredibly good, not just for the woman but for the guy as well.* F

One mother reported being able to use the skills from MBCP while her baby was delivered with a C-section (due to breech position). She reflected on that the practice possibly had increased her ability to step out of negative thought patterns. She explained having feared being numbed by the epidural anaesthesia, but instead of working up an anxiety attack she had managed to let go of those thoughts and focus elsewhere.

### Help with challenges in caring for the baby

For some of the women the establishment of breastfeeding was challenging. Pain from sore nipples was most commonly described. These mothers found that the mindfulness practice helped them relax instead of getting tense, stressed or worried. Having an approach of curiosity combined with reminding oneself that the situation is temporary was also experienced as helpful.

*Nothing is permanent, this is just how it is right now, but there’s no point in thinking about how it was half an hour ago, or about how it will be in an hour ... I had like a foundational approach of curiosity ... Instead of like, reacting with fear.* M

There were also examples of overwhelming situations where nothing seemed to help. A woman described the worry and stress with a baby who was not able to latch properly, and didn’t gain weight like he should during the first couple of weeks. She had been exhausted, had forgotten to sleep and eat and was extremely distressed.

*And in that moment it wasn’t enough with mindfulness or anything, there I didn’t think at all about acceptance or tolerance, I guess it was just tough and I wasn’t able to let go of the worry or learn how to handle it, I was just terribly worried about his weight loss.* M

The participants described that they were more patient in situations of disrupted sleep or with a crying baby thanks to the practice of mindfulness. Nursing the baby during the night became moments of informal meditation and the body scan was used as a method to fall back asleep again after having had to change nappies in the middle of the night. Walking meditation with the baby was used during the night as a way of holding irritation at bay, to stay calm and to easier fall asleep again once the baby had fallen asleep. The parents also perceived the mindfulness practice as broadening their perspective, which was useful during times when the baby was distressed:

*We had one evening when he was quite inconsolable… I guess we took turns walking around with him and calming him down... And at that time I thought about it pretty often when a feeling of upset came up: “No, but take a step back”, and yeah, thinking that “this won’t last forever, it’s just now and then it’s over and then it will be good again and nothing has been lost in the meantime”.* F

The participants described becoming more tolerant, flexible and tuned in with their baby due to the intervention. The expression of tolerance was connected with accepting not having control and remaining calm in stressful situations.

*I guess I think that both the tolerance and the acceptance has increased incredibly much and it has helped. It is super important in this phase of parenting because you can’t steer an infant, you can’t force him into a schedule or make any demands or so. So it’s been a reoccurring thing that you stop and take a deep breath and just realize that ‘No, but we’ll take this in your pace’ … It hasn’t triggered this enormous stress that it would have done otherwise.* M

Mothers described that they shifted toward more informal practice of mindfulness after the birth and how that made them able to see the world with new eyes as well as being more present with the baby. They also experienced that it helped them enjoy peaceful moments and appreciate the nature around them.

*No, but nothing else matters, you can be stressed and worried about other things but you have to put that aside. When you are with the child that is the only thing that counts and it is only here and now.* M

### Relating to each other with more presence and patience

The practice of Insight Dialogue was described as an important learning experience, where participants obtained valuable insights into their communication patterns. The main lessons learned were about the importance of listening. There were participants who thought that this increased capacity to listen might have made it possible for them to find out more about their partner, and thanks to that also be more able to compromise and cooperate. The enhanced skill of being able to listen was also described as being beneficial for the partner, in being listened to. One woman explained that her partner used to have difficulties with expressing himself, but that this had changed after attending the MBCP program. She ascribed this change in part to her own change in behaviour; instead of interrupting him and finishing his sentences she would patiently wait for him to find his own words.

*I can wait for a while. I wait and don’t get irritated … in everyday life … it became easier for him to talk about what he felt and what he was going through. It’s always been easy for me to express my feelings … for him it hasn’t. But now he can, so that was nice.* M

The practices for coping with pain using ice-water and supporting each other were also experienced as strengthening the relationship. This was especially important for a couple who went through some difficulties relationship-wise during the pregnancy, since it helped them to cooperate, talk and feel more supported by each other.

Besides the specific couple-practices mentioned above, the overall attitudinal foundation, including acceptance and letting go, was described as helpful when co-parenting. Participants experienced that the program had helped them be able to accept that the other parent does things differently with the baby, it helped them stop themselves from trying to control everything and instead to trust the other parents competence.

*I can catch myself when my husband is with him that I’m like on my way to tell him about a million of small things all the time, and then I’m like “nah, he can manage this parenthood thing really well” ... You just have to accept that we are different.* M

Learning mindfulness was thus experienced by some of the participants as improving their way of being with each other.

## Discussion

### Summary of the findings

In the present study we have explored participants’ experiences of MBCP. There was a variety in how much the informants perceived MBCP as helpful and variations in their preconceived notions and hopes. Among those who described an active engagement in the course, there were similar patterns in how they experienced improvements in their life. Overall, these improvements were explained as resulting from an increased capacity to focus and from gaining a wider perspective as well as adapting an approach of curiosity, non-judging and acceptance. This enabled new ways of relating to one’s thoughts and emotions. Furthermore, the skills they learned were experienced as helpful for coping with parental challenges such as childbirth, breastfeeding troubles, sleep deprivation and stressful moments with the baby. Finally there were also descriptions of how these new capacities improved some couples’ communication.

Our findings demonstrate that most of the expectant parents experienced MBCP as a valuable preparation for the challenges they met during pregnancy, childbirth and parenthood. Increased skills for coping with stress, anxiety and pain, as well as increased insight, self-compassion and communication were attributed to MBCP by a number of participants. The findings also show that some participants felt that the mindfulness practices did not suit them and that scepticism could be a hindrance for experiencing MBCP as valuable.

### Interpretation in light of the literature

These results are consistent with those of earlier qualitative analyses of MBIs for expectant parents [14, 21–24]. There are also many similarities with results from qualitative studies of other MBIs regarding the idea of acceptance, increased feelings of calm, a change in perspective and an increased understanding both intra- and interpersonally [25–27]. Besides confirming earlier findings, our study also points out that the practice of Insight Dialogue is a valuable part of the MBCP-curriculum. Our study also sheds some light upon how persons who are not interested in mindfulness from the start, and who perhaps never would have signed up for a mindfulness course, can experience it: For some it was of no value, whilst for others it was a surprisingly positive experience. This demonstrates that an open attitude toward mindfulness may be a prerequisite for benefitting from the program, but that scepticism is not a hindrance as long as the participants are curious enough to give the program a chance.

The descriptions of the parents’ experiences of becoming more present in the moment with their baby, more resilient to stress and negative affect and more able to apply a broader perspective and prioritize wisely, supports Duncan et al.’s Model of Mindful Parenting. The model explains that mindfulness enables parents to choose appropriate responses to their child in the moment, since they embody a set of attitudes and insights, rather than a set of skills [28].

The embodiment of mindfulness as our participants expressed it, also ties in to Glück’s and Bluck’s model regarding the development of wisdom [29]. The core elements of their model are the following resources: a sense of mastery, openness, reflectivity, emotion regulation and empathy. These resources help individuals deal with life challenges in a way that makes them grow wiser. ‘A sense of Mastery’ entails an individual’s belief that they are able to deal with life’s challenges as well as being aware of and accepting the inherent uncertainty of human life. This fits with our participants’ descriptions of accepting not being able to control many things and yet exerting control over some areas, such as their attention. This also matches the salutogenic framework by Antonowski, which in part “is to be able to deal with uncertainty and chaos however much we would like to believe we are in control of life” [30].

Our findings show that the participants valued the peer-support and the emotional support in the MBCP-group. This shows that MBCP can possibly fill a gap that Widarsson et al. have pointed out in the Swedish antenatal clinic services–regarding expectant parents’ dissatisfaction with the psychosocial and emotional support and their wish for a forum for expectant parents to meet. [31].

Among our participants, the women were more motivated to participate than the men, which was also the case in the study by Fisher et al. [21]. Regarding motivation it is worth considering Dunn et al’s finding; that motivation for participating in an antenatal MBI is increased if participants have experienced emotional difficulties in the past [23]. This factor possibly mediated our participants’ positive outcomes and might explain the difference in motivation between the pregnant women and their partners, since the study design in the RCT selected pregnant women with an increased risk of stress, anxiety and depression, but the partners were invited to participate simply because they were the partners. However, the benefits of attending the program together as a couple are important. Gambrel and Piercy found for example that some women feel supported simply by the partner agreeing to participate [32]. Our participants, as well as those in Gambrel and Piercy’s study also recognized positive changes in their partner, and it is likely that this strengthened their motivation to engage in mindfulness practices themselves or at least support their partner in doing so.

### Limitations of the study

A weakness of this study is that it didn’t capture the experience of the fathers to the same extent as that the mothers, and we thus do not know if the experience of MBCP was very different for the four fathers who declined to be interviewed. However the data collected does show a large variety of experiences among the six fathers who were interviewed. It is also worth considering that this sample of participants mainly consisted of highly educated and employed persons, which limits the transferability of the findings to parents with fewer resources. However MBIs have been found to be feasible and acceptable by pregnant women with low-income as well [33]. Another possible limitation may be that two of the authors who analyzed the qualitative data are MBCP instructors, which could have biased their analysis. To ensure accuracy and credibility, the analysis was thoroughly discussed with the third independent author.

### Future research

Further studies should explore long-term outcomes of MBCP on parental mental health and child development, using both psychological and physiological measures. An interesting aspect regarding child development is to evaluate MBCPs effect on parent-infant relation and attachment-style. Health-economic calculations to see if the cost of such up-stream intervention can save expenses further downstream are also relevant.

## Conclusions

In order to promote wellbeing among expectant parents it is not enough to only provide them with information (regarding childbirth, breastfeeding and the post-partum period) [28]. Since *inner* resources may go a longer way toward wellbeing than information from the outside, it is preferable that antenatal parent-support programs provide parents with an opportunity to strengthen their inner resources. Our study suggests that MBCP can provide such an opportunity.

## Additional file

Additional file 1: Interview guide, translated. (DOCX 22 kb)

## Abbreviations

MBCP: Mindfulness Based Childbirth and Parenting Program
MBI: Mindfulness Based Intervention
RCT: Randomized Controlled Trial
