# Supplementary material for: What is learned from Mindfulness Based Childbirth and Parenting Education? – Participants’ experiences
Source: BMC Pregnancy Childbirth. 2018 Dec 3;18:466. doi: 10.1186/s12884-018-2098-1 (PMC6276167; doi:10.1186/s12884-018-2098-1)
Supplement: Supplementary file 1 — Interview guide, translated. (DOCX 22 kb) [file 12884_2018_2098_MOESM1_ESM.docx]

**A qualitative study of participants’ experience of MBCP**

Interview guide

First question:

To mothers: How did you experience the time when you were pregnant?

To partners: How did you experience the time when you were expecting the baby?

Follow-up probes (if the participants did not speak about these themes in response to the first question, they were specifically probed for):

*What was is like to participate in the antenatal course?*

*What did you learn? How did it work/ How did you manage?* (regarding that which you learned)

*What was the most important thing you learned? For labour? For parenting an infant?*

*Was there something challenging?*

*Has it affected the way you feel now? Can you describe?*

*Is there something you take with you for the future?*

*Can you give an example of when what you had learned was useful?*

*Can you tell me a little about how you cope with stress/rumination/worries today, compared to before?*

*Has what you have learned affected you everyday life in any way?*

For those who experience that they did not learn anything, ask instead:

*Who do you think could benefit from this kind of course and why?*

*What did you whish to learn?*

Probe further into whether the experience of the course related to:

The birth

*Can you tell me about how you experienced the birth/giving birth?*

*Do you think the course was helpful for giving birth and if so can you tell how?*

*If not, how did you manage?*

*Can you tell me how you coped with an intensive contraction?*

Time as parents to a new-born

*Now when the baby is a few months old, have you experienced any stressful situations? If so, how have you handled them?*

Breastfeeding

*How is it going with the breastfeeding? How have you used your skills from the course for breastfeeding?*

Attachment

*What feelings do you have for your baby?*

*Do you feel that you already understand you baby?*

*How do you and your baby spend time together, what do you do?*

The couple relationship

*How do you think your relationship has developed now that you are parents?*

*Have you kept up the practice of mindfulness? In what way and how often?*

*If not, how come?*
